# Gastric necrosis secondary to gastric volvulus in a paraesophageal hernia: a case report

**DOI:** 10.1093/jscr/rjae072

**Published:** 2024-02-13

**Authors:** Marine Bolliet, Binit Katuwal, Ramachandra Kolachalam

**Affiliations:** Department of Surgery, Ascension Providence Hospital-Michigan State University College of Human Medicine, Southfield Campus, Southfield, MI 48075, United States; Department of Surgery, Ascension Providence Hospital-Michigan State University College of Human Medicine, Southfield Campus, Southfield, MI 48075, United States; Department of Surgery, Ascension Providence Hospital-Michigan State University College of Human Medicine, Southfield Campus, Southfield, MI 48075, United States

**Keywords:** hiatal hernia, paraesophageal hernia, gastric volvulus, gastric necrosis, type IV hiatal hernia

## Abstract

Hiatal hernias are commonly encountered in clinical practice. In certain cases, especially in large hiatal hernias, gastric volvulus can occur. Patients with volvulus typically will present with vomiting, chest pain, shortness of breath, and dysphagia. In extreme cases, gastric volvulus can result in gastric necrosis requiring partial or total gastrectomy. Here we highlight a case of a 76-year-old female with a known large type IV hiatal hernia who was found to have gastric volvulus with necrosis requiring partial sleeve gastrectomy. This case demonstrates the rare, but possible complication of gastric necrosis secondary to gastric volvulus from a large hiatal hernia, prompting emergent surgical intervention.

## Introduction

Gastric volvulus is a rare condition, although no clear incidence has been described in the literature. Approximately 75% of cases of gastric volvulus result from secondary causes such as adhesive disease and hiatal hernias [1]. Hiatal hernias are noted to increase in incidence with age, with people >50 years old having rates >50%, however, only <10% of patients with hiatal hernias are symptomatic [2]. Indications for hiatal hernia repair include any symptomatic hiatal hernia including obstruction and volvulus [3]. In acute gastric volvulus in a paraesophageal hernia, volvulus detorsion is the primary modality of management followed by elective hiatal hernia repair. Detorsion can be achieved with cautious placement of a nasogastric tube, or with endoscopic decompression. In rare circumstances the volvulus can lead to ischemia and necrosis, requiring an emergent surgical intervention also requiring resection.

Gastric necrosis is uncommon due to the robust blood supply of the stomach. Studies have demonstrated that both arterial and venous vasculature must be compromised to lead to gastric ischemia and necrosis [4]. Some causes of impaired blood supply to the stomach include massive dilation due to eating disorders where binge eating is prominent, bowel obstruction, infectious etiologies, intrathoracic herniation, and gastric volvulus [5].

## Case presentation

The patient was a 76-year-old female who was diagnosed with a large paraesophageal hernia 10 years ago and was evaluated 2 years ago for elective repair, but was lost to follow-up. However, she presented acutely with nausea, vomiting, and epigastric/chest pain with computed tomography consistent with gastric volvulus.

**Figure 1:**
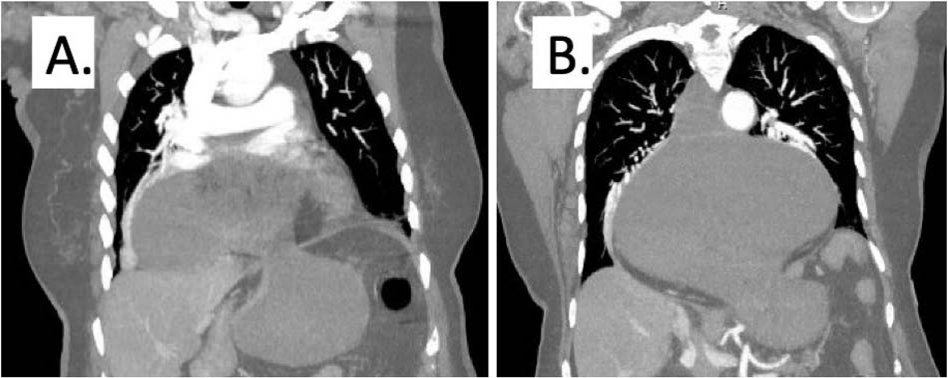
Representative computed tomography image demonstrating gastric distention and area of volvulus (A) and extent of hiatal hernia (B).

On presentation to the hospital, she was noted to have nausea and coffee-ground emesis, and she was complaining of pain in her chest and epigastrium. A computed tomography scan of the chest, abdomen, and pelvis revealed a large hiatal hernia with the gastroesophageal junction in the mid-chest, marked distension of the gastric lumen secondary to volvulus (Fig. 1). The cardiac workup was negative. A nasogastric tube was placed for decompression. Her labs were notable for a lactic acid of 4.1 mmol/L (0.5–2.0 mmol/L) which increased to 4.3 mmol/L even after adequate fluid resuscitation. She developed persistent tachycardia, was febrile to 38.4 C, and began having mental status changes. She was taken emergently to the operating room at this time.

**Figure 2:**
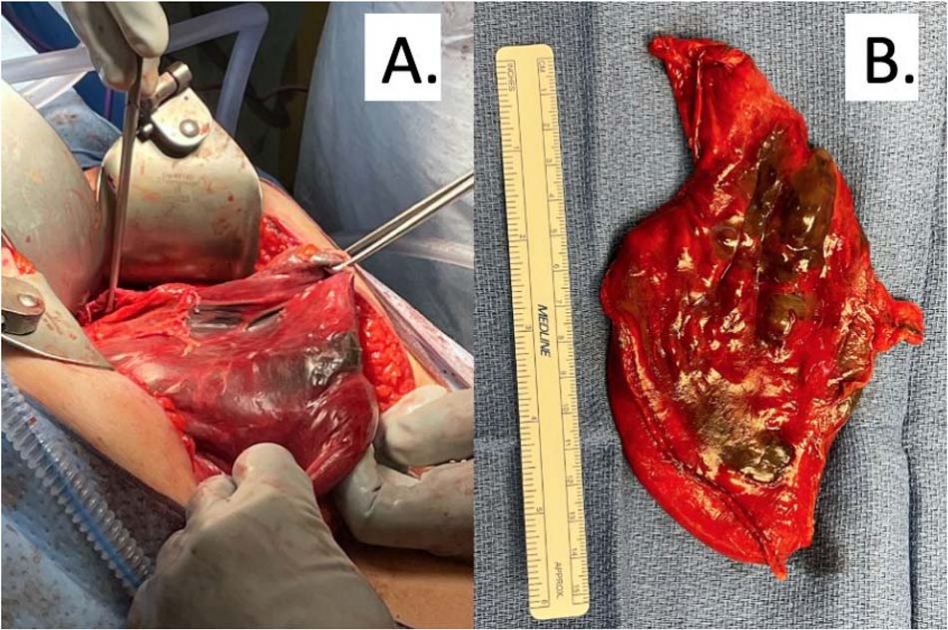
(A) Intraoperative findings of patchy gastric necrosis along greater curvature. (B) Partial gastrectomy specimen with necrotic areas spanning across 10 cm.

In the operating room, an exploratory laparotomy was performed and an incarcerated and entirely intrathoracic stomach with volvulus was noted. Multiple portions of the stomach appeared necrotic, including the body and fundus. Following mediastinal dissection, the hernia was fully reduced into the abdomen and the stomach was fully inspected. A 10-cm area of the greater curvature was found to have patchy necrosis (Fig. 2). This area was resected using an Endo-GIA stapler along the greater curvature up to the gastroesophageal junction, similar to a vertical sleeve gastrectomy. The remaining stomach was re-inspected and found to be viable. The hiatal hernia was repaired with approximation of diaphragmatic crura using three figure-of-eight 0 polyester sutures. Given the extent of the gastric resection, fundoplication could not be performed therefore, a gastropexy to the lateral anterior abdominal wall was performed. Histology of the specimen demonstrated vascular congestion with interstitial hemorrhage and mixed inflammation. Postoperatively she was taken to the intensive care unit and intubated to facilitate ongoing resuscitation and correction of her metabolic acidosis. She was extubated on the second postoperative day and discharged home on postoperative day 11 after conservative management of postoperative ileus. On follow-up, she was noted to be doing well, tolerating a liquid diet, and was advanced to a soft diet. She had no reflux symptoms and her incisions were healing well.

## Discussion

Hiatal hernias have a prevalence ranging from 10 to 50% [6]. A great majority of patients with hiatal hernias are asymptomatic and most are found incidentally. Treatment options for symptomatic type IV hiatal hernias are primarily surgical, with medical treatment being an option in patients who are at high surgical risk. Long-standing hiatal hernia complications include gastritis, reflux, esophagitis, esophageal dysplasia, obstruction, and respiratory symptoms secondary to compression [7]. In extreme circumstances, gastric volvulus can result, leading to ischemia and, in rare cases, to necrosis requiring surgical resection.

Initial management of gastric volvulus includes nasogastric decompression, fluid resuscitation, and close monitoring. There is a role for early endoscopy to evaluate for mucosal ischemia as well as for endoscopic reduction of the volvulus [8]. In our case, plans for endoscopic intervention were overruled by clinical deterioration requiring emergent surgical intervention.

There are very few cases that describe gastric necrosis requiring resection secondary to volvulus, with most being seen in the pediatric population [9–11]. The stomach is a resilient organ that has been shown to resist necrosis even in circumstances where significant arterial supply is compromised, but in cases where both arterial supply and venous drainage have been affected the risk of necrosis is high [4].

With regards to options for repair, all interventions must include hernia reduction and repair in addition to detorsion of the stomach. Resection is reserved for necrosis. There has been a debate on whether antireflux surgery is included with the hiatal repair. However, some authors do believe that fundoplication is beneficial after hiatal hernia repair to limit symptoms of reflux and to provide additional support to the repair [3, 11, 12]. There is extensive discussion about the role of performing gastropexy without formal hernia repair with data demonstrating high recurrence rates of up to 17% [1].

## Conclusion

Gastric volvulus is a known complication secondary to large hiatal hernias. In cases where patients have a known hiatal hernia and present with acute onset epigastric/chest pain, a high index of suspicion should be maintained due to the possibility of gastric volvulus. In the majority of gastric volvulus cases, the robust blood supply of the stomach makes it resistant to ischemia and necrosis. As a result, detorsion often is sufficient to restore blood supply. In rare cases, the ischemia can lead to perforation or necrosis requiring resection. Due to this increased risk of volvulus, large hiatal hernias should be repaired electively.

## Data Availability

Data sharing is not applicable to this article as no datasets were generated or analyzed during the current study.
